# Pharmacokinetics of ferric pyrophosphate citrate administered via dialysate and intravenously to pediatric patients on chronic hemodialysis

**DOI:** 10.1007/s00467-018-4014-3

**Published:** 2018-07-12

**Authors:** Raymond D. Pratt, Sarah Grimberg, Joshua J. Zaritsky, Bradley A. Warady

**Affiliations:** 1 Rockwell Medical, Inc., 30142 Wixom Rd, Wixom, MI USA; 2 Department of Pediatrics, Nemours/Alfred I. duPont Hospital for Children, Wilmington, DE USA; 3 Department of Pediatrics, Children’s Mercy Kansas City, Kansas City, MO USA

**Keywords:** Ferric pyrophosphate citrate, Hemodialysis, Iron, Pediatric, Pharmacokinetics

## Abstract

**Background:**

Iron deficiency is a common cause of anemia in pediatric patients with hemodialysis-dependent chronic kidney disease (CKD-5HD). Ferric pyrophosphate citrate (FPC, Triferic®) donates iron directly to transferrin, bypassing the reticuloendothelial system and avoiding iron sequestration. Administration of FPC via dialysate or intravenously (IV) may provide a suitable therapeutic option to current IV iron preparations for these patients.

**Methods:**

The pharmacokinetics and safety of FPC administered via dialysate and IV to patients aged < 6 years (*n* = 3), 6 to < 12 years (*n* = 4), and 12 to <18 years (*n* = 15) were investigated in a multicenter, open-label, two-period, single-dose study. FPC (0.07 mg iron/kg) was infused IV into the venous blood return line during hemodialysis session no. 1. FPC iron was added to bicarbonate concentrate to deliver 2 μM (110 μg/L) iron via dialysate during hemodialysis session no. 2.

**Results:**

Mean serum total iron concentrations peaked 3 to 4 h after administration via dialysate and 2 to 4 h after IV administration and returned to baseline by 10 h after the start of hemodialysis for both routes. Iron exposure was greater after administration via dialysate than after IV administration. The absolute amount of absorbed iron after administration via dialysate roughly doubled with increasing age, but the weight-normalized amount of absorbed iron was relatively constant across age groups (~ 0.06–0.10 mg/kg). FPC was well tolerated in the small number of patients studied.

**Conclusions:**

FPC iron can be administered to pediatric patients with CKD-5HD via dialysate or by the IV route. Further study of FPC administered to maintain hemoglobin concentration is indicated.

## Introduction

The goals of iron therapy in hemodialysis-dependent chronic kidney disease (CKD-5HD) patients are to avoid depletion of iron stores, prevent iron-restricted erythropoiesis, and maintain target hemoglobin levels while avoiding blood transfusions that may sensitize patients and limit chances for a kidney transplant [1]. Both erythropoiesis-stimulating agents (ESAs) and iron are essential components for this management. Utilization of current intravenous iron products requires uptake and metabolism in the reticuloendothelial system (RES) via the red blood cell salvage pathway to export ferrous iron. Subsequent to its oxidation to ferric iron, it is bound to circulating transferrin [2]. Systemic inflammation in patients with CKD-5HD results in an elevation of hepcidin, which blocks absorption of iron across the enterocyte and impairs export of iron from the RES [3, 4]. The block to macromolecular iron utilization and the use of IVFe as “maintenance therapy” has contributed to a substantial rise in serum ferritin levels in the chronic dialysis population [5–8]. This functional iron deficiency (FID) and rise in ferritin has led to concerns that IV iron may produce iron overload and contribute to the inflammation, tissue injury, endothelial dysfunction, and oxidative stress that has been described with the use of these agents [8, 9].

Ferric pyrophosphate citrate (FPC, Triferic®) is a novel complex iron salt that is water soluble and contains no carbohydrate shell. FPC is approved in the USA as an iron-replacement treatment to maintain hemoglobin concentrations in adult patients with CKD-5HD. FPC is added to the bicarbonate concentrate at each hemodialysis session. The bicarbonate concentrate, with FPC iron, mixes with acid concentrate and water in the hemodialysis machine to generate dialysate containing 2 μM (110 μg/L) of iron (III). FPC crosses the dialyzer membrane and donates iron directly to transferrin. Transferrin bound iron is rapidly cleared from the circulation [10]. This provides iron for erythropoiesis and bypasses the hepcidin-induced iron sequestration within reticuloendothelial macrophages [11, 12].

The FPC dose of 2 μM iron in the dialysate was optimized for adult patients who have high dialysate and blood flow rates and dialysis membranes with large surface areas. FPC (molecular weight, ~ 1300 Da) diffuses across the dialyzer membrane at a rate comparable to that of vitamin B_12_ and results in an intradialytic increase in serum total iron (sFe) of approximately 100 μg/dL [13–15]. Preliminary studies of FPC administered IV in adult HD patients using a pharmacokinetic approach established that 6.5 mg of FPC is administered via dialysate at each HD session [16]. When scaled on a milligrams per kilogram basis, the IV dose is approximately 0.13 mg Fe/kg. The current study was conducted to evaluate the pharmacokinetics and preliminary safety of FPC and to evaluate the dose of FPC administered via the dialysate to pediatric patients with CKD-5HD. The study also examined the feasibility of IV administration of FPC in pediatric patients. Intravenous administration of FPC would allow patients whose HD systems do not use liquid bicarbonate concentrate to receive FPC iron, thus potentially providing a suitable therapeutic option to the currently available IV iron preparations.

## Methods

The study was conducted in accordance with the Declaration of Helsinki and Good Clinical Practice guidelines. The protocol and informed consent and assent documents were approved by an institutional review board for each participating site before any patients were enrolled in the study at the respective site. Written informed consent was obtained from each patient’s parent or legal guardian and, if applicable, assent was obtained from the patient before any study-related procedures were performed. The study was registered at www.clinicaltrials.gov as NCT02595437.

### Patients

Patients between 1 and ≤ 18 years with CKD-5HD who had received chronic hemodialysis for ≥1 month, had a vascular access suitable to support blood flows for HD, were adequately dialyzed (Kt/V > 1.2), and had a hemoglobin concentration ≥ 10.0 g/dL, a transferrin saturation (TSAT) ≥20%, and a serum ferritin concentration > 100 μg/L were enrolled. Exclusion criteria included concomitant infection, active inflammatory disorder, body weight < 5 kg, use of IV or oral iron supplements ≤ 2 weeks before baseline, and changes in ESA dose ≤ 3 weeks before baseline. Female patients were prepubertal or practicing adequate birth control. There were no upper limit exclusions for hemoglobin or ferritin concentrations. Negative pregnancy tests were obtained before the first FPC treatment for females aged ≥ 9 years.

### Study design and drug administration

This was a multicenter, open-label, two-period, single-dose study of the pharmacokinetics and safety of FPC administered IV and via dialysate to pediatric patients on chronic HD. The study was planned to include up to a total of 24 patients aged < 6 years (2–4 patients), 6 to < 12 years (6–8 patients), and 12 to < 18 years (12–16 patients). The study included a screening period of up to 2 weeks to establish study eligibility, and two FPC treatment sessions in sequential order: (1) a single 0.07-mg iron/kg dose of FPC iron in 100 mL of dextrose 5% water (D5W) as a continuous IV infusion into the venous blood return line beginning at the start of and continuing for the duration of the HD session and (2) FPC added to the bicarbonate concentrate to deliver a final dose of 2 μM (110 μg/L) iron via the dialysate over the duration of the HD session. To ensure patient safety, the initial IV dose of FPC administered to pediatric HD patients was decreased by half from the adult dose to ensure that iron binding capacity was not exceeded. The first FPC treatment was administered at the second HD session, and the second FPC treatment was administered at the third HD session of the week for patients who dialyzed three times per week or at the first and second HD session for patients who dialyzed two times per week. Hemodialysis sessions began in the morning, started at the same clock time, and was of the same duration for a given patient. The sequence of administration was based on the adult experience with FPC which showed no sequence effects of dialysate or IV administration on FPC iron pharmacokinetic parameters.

FPC was supplied by Rockwell Medical, Inc. (Wixom, MI) as a liquid concentrate formulation of 27.2 mg of FPC iron per vial (5.44 mg Fe/mL). The IV dosing solution was prepared by adding 3.2 mL of the FPC solution to 500 mL of D5W to give a final concentration of 0.035 mg/mL of iron in the stock solution. The final volumes of stock solution and diluent were adjusted based on the patient’s weight to achieve a dose of 0.07 mg iron/kg/HD to be administered during the HD session. The FPC iron dialysate solution was prepared by adding one 5-mL ampule of FPC to 2.5 gal of liquid bicarbonate concentrate or 2 mL FPC concentrate per gallon of pre-packaged liquid bicarbonate concentrate and mixing by inverting the container. The final concentration of iron in the dialysate was 2 μM (110 μg Fe/L) when used in a 45× dialysis machine.

### Study assessments

Serial blood samples for pharmacokinetic assessments of sFe and transferrin-bound iron (TBI) were obtained before and at 1, 2, 4 (or end of HD), 4.5, 5, 6, 8, and 10 h after the start of HD. Blood samples for a safety serum iron profile (sFe, ferritin, TSAT, and total iron-binding capacity [TIBC]) were collected before and at 4 h after the start of each HD session. Vital signs were measured before, every 30 min during, and within 30 min after the end of HD. Adverse events were recorded as observed or reported.

### Bioanalytical methods

Serum samples were analyzed for sFe and free iron (i.e., non-transferrin-bound iron [NTBI]) at QPS Laboratories (Groningen, The Netherlands) using validated assays. Acid digestion was used to extract sFe from the serum samples. Extraction of NTBI was performed by ultrafiltration of the serum samples, followed by acid digestion. Inductively coupled plasma-mass spectrometry (ICP-MS) was used to measure sFe and NTBI concentrations in serum. Transferrin concentration was measured using a validated enzyme-linked immunosorbent assay (ELISA).

### Pharmacokinetic and safety analyses

Concentrations of sFe and NTBI and pharmacokinetic parameters for sFe were summarized using baseline-corrected values to account for variability among patients in sFe concentrations before administration of FPC. Pharmacokinetic parameters of sFe were calculated using standard noncompartmental pharmacokinetic analyses in Phoenix® WinNonlin® (version 6.3, Certara, Princeton, NJ). The concentration of TBI in serum was calculated as the difference between sFe and NTBI.

Pharmacokinetic parameters and continuous safety parameters were summarized with descriptive statistics. Categorical safety parameters were summarized with counts and percentages. No formal statistical analyses were performed on safety parameters. All analyses were performed using SAS statistical software, version 9.1.3 or higher (SAS Institute, Inc., Cary, NC).

## Results

### Patient demographics and disposition

A total of 22 patients (11 male and 11 female), mean age 11.8 years, were enrolled in the study: 3 patients were < 6 years, 4 patients were 6 to < 12 years, and 15 patients were 12 to <18 years. The demographic characteristics and renal history of the patients were consistent with the age groups under study (Table 1). Across all age groups, the patients were primarily white (36%) or black/African-American (36%) and not of Hispanic nor Latino ethnicity (55%). Mean time since initial diagnosis of chronic kidney disease (CKD) was 2.9 years, and mean time since first dialysis was 32.2 months for all patients. The median time since the first HD was 17.6 months for all patients and ranged from 8.9 months for patients < 6 years, to 23.5 months for patients 12 to < 18 years, to 28.2 months for patients 6 to < 12 years. Mean dialyzer surface area was 1.4 m^2^ for all patients. Mean dialyzer surface area increased with increasing age. All patients in each age group were iron replete at baseline (TSAT ≥ 20% and serum ferritin level > 100 μg/L). Mean baseline hemoglobin concentration was 11.5 g/dL, with a range of 10.3 to 13.7 g/dL. A few patients were enrolled with a hemoglobin concentration > 12.5 g/dL because they met all other inclusion criteria and had the hemoglobin concentration that their physician had targeted. There were no clinically relevant differences among age groups in screening and post-study clinical chemistry (Chemistry 20 analysis) and hematology (CBC) assessments.

**Table 1:** Demographic characteristics and renal history at baseline

Variable^a^	Age group			All patients (*N* = 22)
	< 6 years (*N* = 3)	6–< 12 years (*N* = 4)	12–< 18 years (*N* = 15)	
Sex				
Male	2 (67%)	1 (25%)	8 (53%)	11 (50%)
Female	1 (33%)	3 (75%)	7 (47%)	11 (50%)
Age (years)	1.7 (1.2)	9.0 (2.2)	14.5 (1.9)	11.8 (5.0)
Race				
White	0 (0%)	0 (0%)	8 (53%)	8 (36%)
Black or African-American	2 (67%)	2 (50%)	4 (27%)	8 (36%)
Other	1 (33%)	2 (50%)	3 (20%)	6 (27%)
Ethnicity				
Not Hispanic or Latino	3 (100%)	3 (75%)	6 (40%)	12 (55%)
Hispanic or Latino	0 (0%)	1 (25%)	8 (53%)	9 (41%)
Unknown	0 (0%)	0 (0%)	1 (7%)	1 (5%)
Baseline predialysis body weight (kg)	12.6 (5.0)	27.2 (9.4)	49.0 (15.5)	40.1 (19.3)
Height (cm)	88.1 (13.6)	126.8 (20.3)	152.1 (12.0)	138.8 (26.3)
Time since initial diagnosis of renal failure (years)	2.5 (1.4)	5.5 (4.7)	2.5 (1.9)	2.9 (2.5)
Time since first dialysis (months)	30.1 (15.9)	45.9 (38.8)	29.0 (22.5)	32.2 (24.9)
Time since first HD (months)^b^	8.9	28.2	23.5	17.6
Dialyzer surface area (m^2^)	0.6 (0.2)	1.0 (0.4)	1.6 (0.3)	1.4 (0.5)
Baseline hemoglobin (g/L)	116 (7.5)	119 (14.9)	113 (7.0)	115 (8.7)
Baseline ferritin (ng/mL)	169 (104.1)	404 (268.6)	410 (380.1)	373 (333.9)
Baseline TSAT (%)	20 (3.1)	22 (12.8)	32 (15.1)	28 (13.9)
Baseline TIBC (μg/dL)	270 (46.8)	239 (29.4)	274 (79.8)	266 (67.0)

*HD* hemodialysis, *TIBC* total iron-binding capacity, *TSAT* transferrin saturation

^a^Values are reported as mean (SD) unless otherwise indicated

^b^Median

Of the 22 enrolled patients, 21 received both the IV dose of FPC and FPC via the dialysate and completed the study. The remaining patient (12- to < 18-year age group) withdrew consent to participate in the study (because of the time commitment for the study) 5 min after the start of the IV infusion of FPC during HD session no. 1 and, therefore, did not receive FPC via the dialysate.

### Pharmacokinetics

**Fig. 1:**
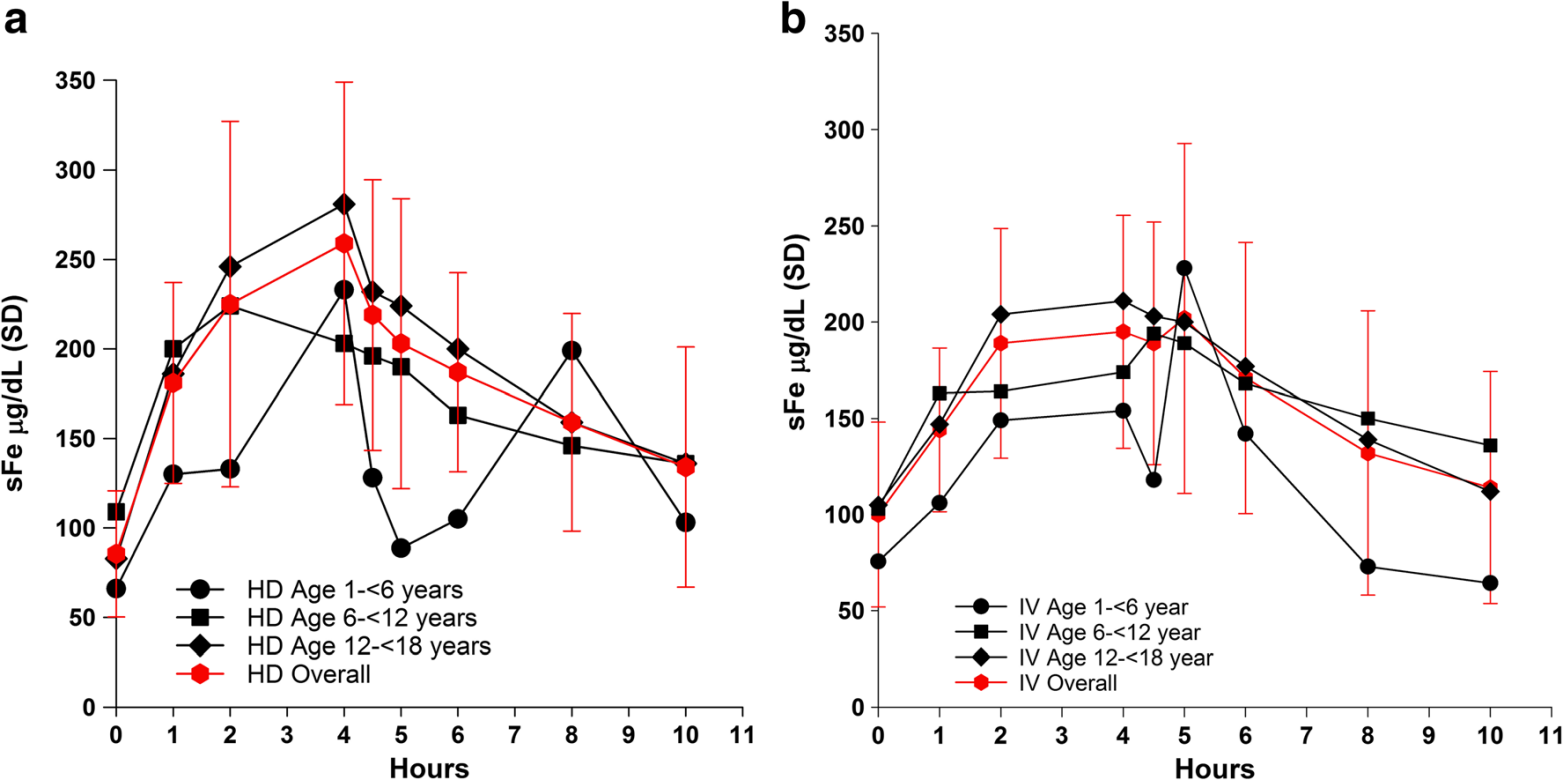
Mean concentration-time plots for serum total iron (sFe) after administration of ferric pyrophosphate citrate (FPC) via hemodialysis (HD) at a concentration of 2 μM (110 μg/L) iron (**a**) and after intravenous (IV) administration of 0.07 mg Fe/kg of FPC by age group (years) and overall (**b**)

**Fig. 2:**
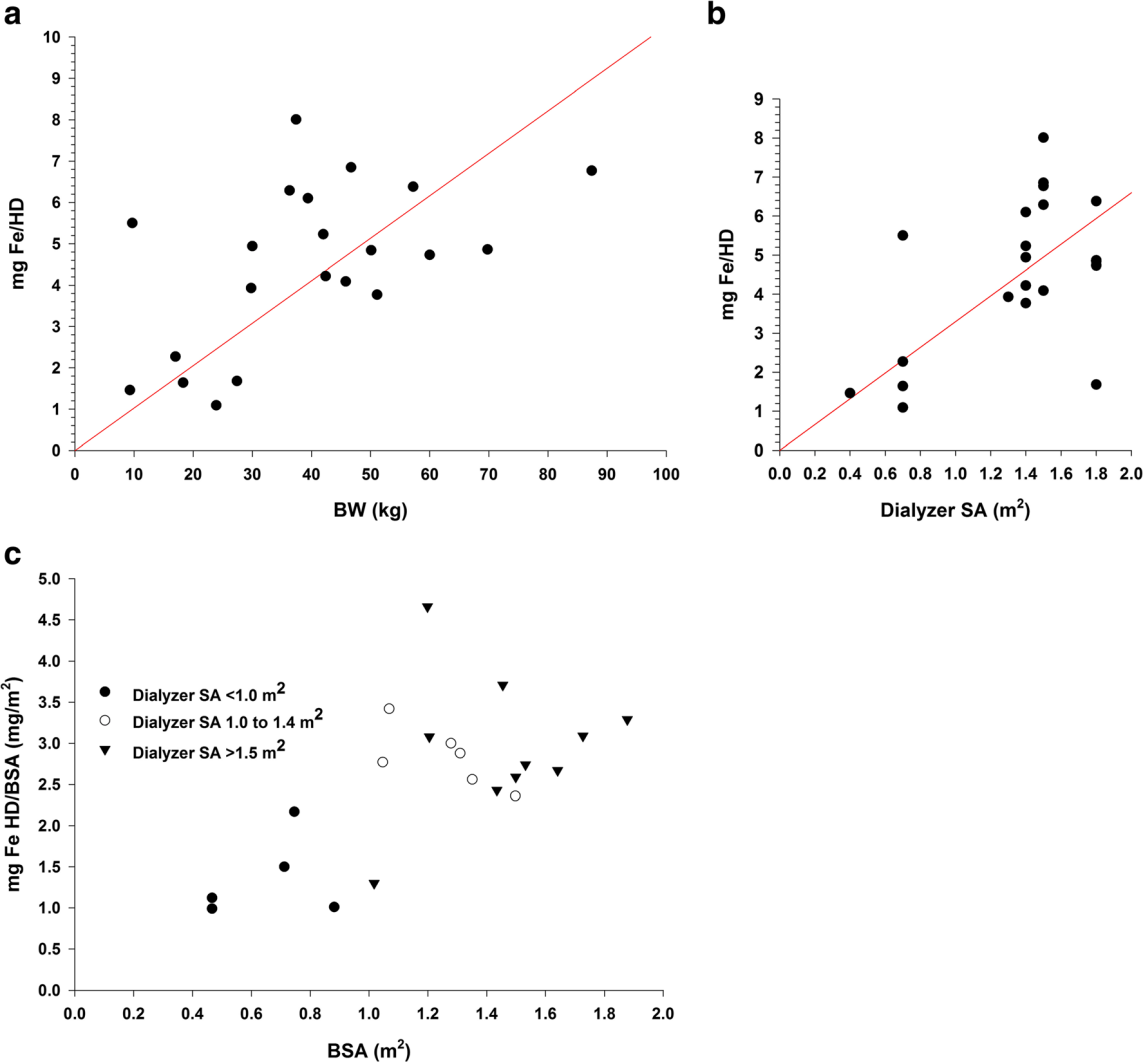
Amount of iron (Fe) administered during hemodialysis (HD) by predialysis body weight (BW) (**a**) by dialyzer surface area (SA) (**b**) and by body surface area (BSA) (**c**): filled circle, Dialyzer SA < 0.8 m^2^; open circle, Dialyzer SA 0.8 to 1.4 m^2^; filled inverted triangle, Dialyzer SA >1.4 m^2^

Mean sFe concentrations peaked at 3 to 4 h after administration of FPC via dialysate (Fig. 1a) and at 2 to 4 h after IV administration of FPC (Fig. 1b). Concentrations of sFe returned to baseline levels by approximately 10 h after the start of HD for both routes of administration. Concentrations of sFe were generally higher in the 12- to < 18-year age group than in the < 6- and 6- to < 12-year age groups. Older patients received a proportionately greater dose of iron via dialysate than the younger patients because of larger dialyzer surface areas for these patients than for the dialyzers used by patients < 12 years of age (i.e., the amount of iron delivered via dialysate was primarily related to the surface area of the dialyzer) (Fig. 2a, b). Body surface area (BSA)-normalized FPC iron delivery via HD (Fig. 2c) shows that there are multiple factors related to body size that impact the apparent iron delivery including membrane surface area, body weight, and BSA. The net result is that there is lower BSA-normalized iron delivery for younger patients compared to older patients.

**Fig. 3:**
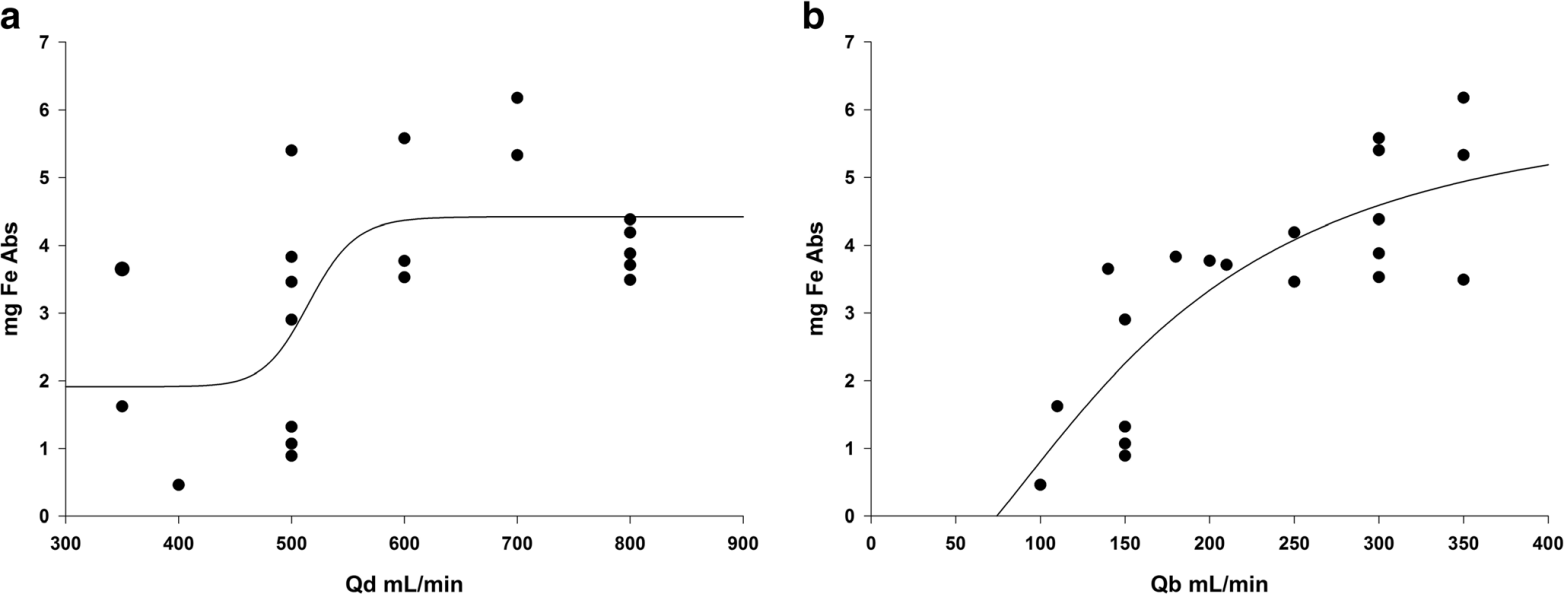
Amount of iron (Fe) administered during hemodialysis (HD) by dialysate flow rate (Qd) (**a**) and blood flow rate (Qb) (**b**). Regression lines are fitted by nonlinear regression in SigmaPlot V14.0

Dialysate flow rate (Qd) had a small effect on the delivery of iron (Fig. 3a), although the small number of patients evaluated precluded a more rigorous analysis. Blood flow rate (Qb) showed increasing dialysance of FPC iron approaching a plateau at 300 mL/min (Fig. 3b). This is consistent with the dialysance properties of molecules with molecular weight of 1000 to 2000 Da (i.e., vitamin B12).

**Fig. 4:**
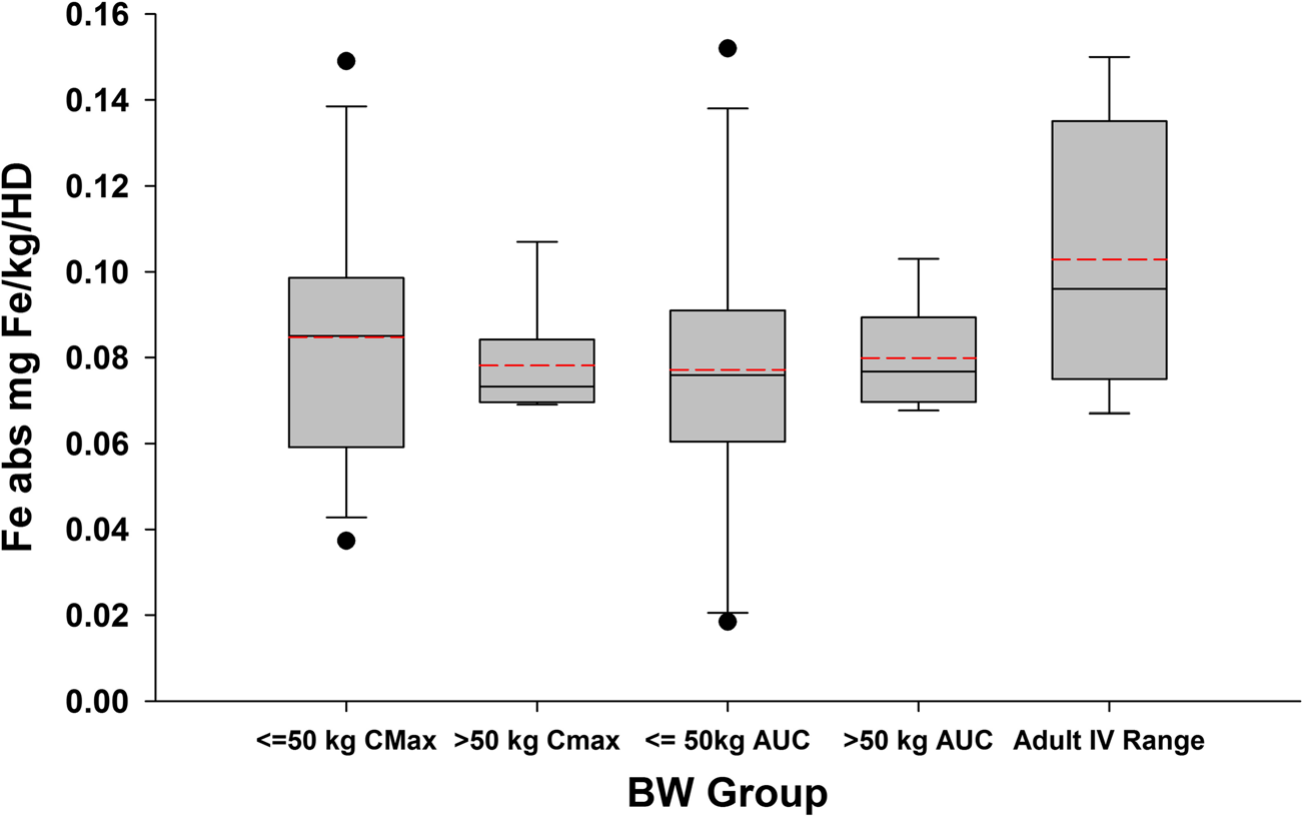
Box plot of weight-normalized absolute iron (Fe abs) administration via hemodialysis (HD) by predialysis body weight (BW) group (≤ 50 and > 50 kg), as calculated by maximum serum iron concentration (*C*_max_) and area under the serum concentration-time curve from time zero to the time of the last quantified concentration (AUC). The last box indicates the estimated weight-normalized doses that were observed in adults who received ferric pyrophosphate citrate (FPC) via HD in phase 3 clinical trials based on pre- to post-HD serum iron concentrations and equivalence to administration of Triferic 6.75 mg IV in equivalence trials. The solid line represents the median dose, the red dash line represents the mean, the error bars show the 95% CI, and the solid dots show the outliers

Iron exposure (based on maximum serum concentration [*C*_max_] and area under the serum concentration-time curve from time zero to the time of the last quantified concentration [AUC_last_]) was greater after administration of FPC via dialysate than after IV administration for all patients (Table 2). The absolute amount of iron absorbed after administration of FPC via the dialysate roughly doubled with increasing age (i.e., body weight; Fig. 2a); however, there was considerable variation in the administered dose. The weight-normalized amount of iron administered via dialysate was approximately 0.06 to 0.10 mg/kg (Fig. 4).

**Table 2:** Baseline-corrected noncompartmental pharmacokinetic parameters of serum total iron in pediatric patients after administration of ferric pyrophosphate citrate intravenously and via dialysate

Route of administration	Pharmacokinetic parameter^a^		
	*C*_max_ (μg/dL)	AUC_last_ (h μg/dL)	*t*½ (h)
Intravenous (*N* = 21)	114 (53.7)	419 (101.6)	1.60 (190.1)^b^
Via dialysate (*N* = 20)	166 (54.3)	682 (82.9)	1.98 (60.6)^c^

*AUC*_*last*_ area under the serum concentration-time curve from time zero to the time of the last quantified concentration, *C*_*max*_ maximum drug concentration in serum, *CV%* percent coefficient of variation, *t½* terminal phase half-life

^a^Values are reported as geometric mean (geometric CV%)

^b^*n* = 8

^c^*n* = 10

The change in serum transferrin concentration between the start and end of the HD session was relatively minor (~ 14%), indicating that the change in volume due to the HD session had only minor impacts on the pharmacokinetics of sFe. NTBI did not exceed 1% of sFe in any sample. Separate analyses of TBI were not performed because sFe was 99% of TBI, supporting direct donation of FPC iron to transferrin.

### Safety

No deaths or serious adverse events were reported, and administration of FPC was not interrupted or discontinued in any patient because of adverse events. Treatment-emergent adverse events were reported in 6 of the 22 patients (27%) (Table 3). All adverse events were reported at a low incidence and were generally mild in severity and not attributed to FPC. Only one drug-related event (axillary pain of moderate severity) was reported.

**Table 3:** Treatment-emergent adverse events after administration of ferric pyrophosphate

Preferred term	Age group			All Patients (*N* = 22)
	<6 years (*N* = 3)	6- < 12 years (*N* = 4)	12- < 18 years (*N* = 15)	*n* (%)
	*n* (%)	*n* (%)	*n* (%)	
At least 1 treatment-emergent adverse event	0 (0)	2 (50)	4 (27)	6 (27)
Nausea	0 (0)	0 (0)	2 (13)	2 (9)
Abdominal pain	0 (0)	0 (0)	1 (7)	1 (5)
Abdominal pain upper	0 (0)	0 (0)	1 (7)	1 (5)
Axillary pain	0 (0)	0 (0)	1 (7)	1 (5)
Back pain	0 (0)	0 (0)	1 (7)	1 (5)
Constipation	0 (0)	1 (25)	0 (0)	1 (5)
Dizziness	0 (0)	0 (0)	1 (7)	1 (5)
Dyspnea	0 (0)	1 (25)	0 (0)	1 (5)
Hypotension	0 (0)	1 (25)	0 (0)	1 (5)
Tachycardia	0 (0)	1 (25)	0 (0)	1 (5)

The safety iron profile of FPC was consistent with the administration of an iron-replacement product, with increases from baseline in sFe, TSAT, and serum ferritin observed at the end of HD both after IV administration and after administration via dialysate. Values for TSAT exceeded 100% (ranging from 102–115%) at the end of HD in two patients in the 12- to < 18-year age group after IV administration of FPC and in one patient in the 6- to < 12-year age group and three patients in the 12- to < 18-year age group after administration of FPC via dialysate. No adverse events were associated with the slight oversaturation of TSAT in these patients. No clinically relevant effects of FPC were observed on systolic or diastolic blood pressure or pulse rate.

## Discussion

The results of this study demonstrate that the pharmacokinetic characteristics of FPC after administration to pediatric patients with CKD-5HD are the same as those observed in adult patients with CKD-HD [11] and in healthy volunteers [13]. The IV dose of FPC (0.07 mg iron/kg) was a reference dose chosen to provide a modest dose of iron to raise the TSAT from a baseline of approximately 25% to a peak of 50 to 60%. The dose of FPC iron administered during HD via the dialysate was, as expected, larger than the dose administered via IV infusion. The amount of iron transferred to patients via the dialysate was proportional to the dialyzer surface area.

FPC was designed as a maintenance iron therapy and not as a repletion treatment. The strategy is to provide small doses of iron that are immediately bioavailable (i.e., bound to transferrin) and rapidly delivered to iron-requiring tissues such as the reticulocytes. FPC is unlike other macromolecular parenteral iron products that are indicated as repletion treatments. These products contain iron surrounded by a carbohydrate shell to minimize the likelihood that NTBI (labile plasma iron) will be present to induce oxidative stress. FPC iron is present as TBI if the serum (plasma) iron concentration is below the TIBC. Transient excursions of FPC iron above 100% TSAT may result in small amounts of iron cleared via NTBI pathways; however, the rapid overall clearance minimizes the cellular uptake via these mechanisms.

The results of this study support continued development of FPC as a maintenance iron therapy for pediatric patients. In adult patients, delivery of FPC iron via the dialysate maintained iron balance and hemoglobin concentrations for up to 1 year, without increasing body iron stores, as measured by ferritin concentrations [14, 15]. This corresponds to an iron delivery of approximately 6.75 mg at each HD session or approximately 1050 mg iron/year. This amount is within the estimated iron losses from the London Daily/Nocturnal Hemodialysis Study [17] and by estimates of residual blood in the dialyzer circuit [18, 19]. Few studies have examined blood (iron) losses in pediatric HD. An early study estimated losses to be between 5 and 8.4 mg Fe/HD [20]. However, the study was conducted using older parallel plate dialysis technology (1977) and, therefore, likely overestimates blood losses using modern hollow fiber dialyzers. Another study estimated iron losses in pediatric HD to be approximately 200 to 300 mg/year, primarily from dialysis, phlebotomy, and gastrointestinal losses [21]. Current guidelines suggest weekly to biweekly does of IV iron products [1] to reduce the dose of ESA needed to maintain a target hemoglobin concentration. However, this regimen may result in iron overload, as macromolecular iron is first processed in the RES, with elevated ferritin concentrations comparable to those observed in adult patients. Mean serum ferritin concentrations in this study were 282.1 ng/mL in the < 6-year age group, 426.3 ng/mL in the 6- to 12-year age group, and 506.9 ng/mL in the 12- to < 18-year age group. The range of individual serum ferritin levels in the pediatric patients in this study (109.4–1712 ng/mL) was comparable to the concentrations that are observed in adult HD patients.

Clinical practice of pediatric HD differs from that of adult patients. Most pediatric HD centers are hospital based and have many fewer patients than a typical adult center. Because of the smaller patient load, many pediatric centers do not mix liquid bicarbonate on site and rely on solid bicarbonate cartridges or premixed liquid bicarbonate in containers to provide the concentrated bicarbonate to the dialysis machine.

This study has demonstrated that Triferic can be administered via the addition to liquid bicarbonate concentrate (dialysate) or IV to pediatric HD patients, resulting in similar amounts of iron administration. The weight-normalized doses of FPC iron via HD in this study (Fig. 3) ranged from 0.06 to 0.1 mg iron/kg. Smaller patients had lower overall exposures, but clearance remained the same as in heavier patients.

When compared with the range of weight-normalized iron per administration in adults in the phase 3 clinical trials (Fig. 4), the amount of iron provided per administration to the pediatric patient is similar. Based on these results, this study supports IV administration of FPC at an estimated starting dose of 0.1 mg iron/kg body weight in patients who weigh < 50 kg (dose range, 1–5 mg Fe/HD). This should achieve a post dialysis TSAT of approximately 75%. The adult dose of 6.75 mg IV should be administered to patients who weigh > 50 kg. These doses should approximately match ongoing blood losses; however, additional supplementation of iron may be required to support tissue growth. Because the iron requirements of pediatric HD patients have not been definitively determined, further investigations of FPC for maintaining hemoglobin concentration are needed and are planned.

The use of IV or dialysate administered FPC will provide the flexibility to centers to provide a suitable maintenance iron regimen to maintain hemoglobin concentrations without increasing body iron stores. When patients become iron deficient, standard IV iron therapy can be used for repletion of iron stores while continuing to provide maintenance iron via FPC.

The safety profile of FPC in this study indicates that FPC is well tolerated, with no treatment-emergent adverse events attributable to FPC. These findings are consistent with findings in adults in who over 650,000 individual doses of FPC have been administered with no reports of anaphylaxis or other adverse effects (data on file, Rockwell Medical, Inc.).

The limitations of this study are the small number of patients studied and the requirement that patients meet criteria for clinical stability at inclusion. At the same time, the study enrolled a wide age range of patients (from ages 1 to 18 years) allowing preliminary assessment of FPC PK in all age groups that receive chronic hemodialysis. While the safety profile of FPC in this study is similar to adults, a larger study population is required to firmly establish the safety profile in pediatric HD patients.

## Conclusions

FPC iron can be administered to pediatric patients with CKD-5HD either via the dialysate (if dialyzer uses liquid bicarbonate concentrate) or IV (either if dialysis machine uses a solid bicarbonate cartridge/bag system or for physician preference). The pharmacokinetics of FPC in pediatric patients are consistent with the pharmacokinetics in adult patients with CKD-5HD and healthy volunteers. FPC was well tolerated by pediatric patients, with no new safety findings. The recommended initial dose of FPC for future studies in pediatric patients with CKD-5HD is 2 μM (110 μg iron/L) in dialysate or 0.1 mg iron/kg IV during HD, using weight-based dosing for patients weighing < 50 kg and 6.75 mg IV for patients weighing > 50 kg.
